# Opening the Bite with Cap-Fillings, Preserving the Vitality of the Pulp

**Published:** 1897-12

**Authors:** M. F. Finley

**Affiliations:** Washington, D. C.


### Opening the Bite with Cap-Fillings, Preserving the Vitality of the Pulp.

BY M. F. FINLEY, D.D.S., WASHINGTON, D. C.

Abstract of paper read at American Dental Association, Old Point Comfort, Va., August, 1897.

In the case described the patient, a male aged 62 years, had every tooth present in the upper jaw, and all in regular position, except the left cuspid, which closed inside. The teeth were so peculiarly worn that when the jaws were closed the superior incisors came in contact with and pressed upon the lower gums, the incisive edges of the lower incisors also impinging on the upper gums when the teeth were closed. The outer cusps of the upper molars also came in close contact with the gums of the lower jaw, forcing the food into painful contact with the gums, causing great discomfort. The molars being sound, with living pulps, it was decided to open the bite by means of cap-fillings upon the second bicuspid and first and second molars on the left side lower jaw, the second bicuspid and third molar right lower heavy crowned to carry a bridge to replace the lost first and second molars, the occlusion being raised to correspond with the opened bite.

The occlusal surfaces of the teeth to be capped were ground to nearly a plane surface and caps adjusted by means of platinum pins, entering four holes in the molars and three in the bicuspids, very carefully located in the centers of the sides, avoiding the cornua. The caps and bridge were all cemented to place at one time to avoid irritation by pressure of a single tooth or on one side of the jaw.

Models, photos and similar cap-filling accompanied the paper.

